# Fossil basicranium clarifies the origin of the avian central nervous system and inner ear

**DOI:** 10.1098/rspb.2022.1398

**Published:** 2022-09-28

**Authors:** Luis M. Chiappe, Guillermo Navalón, Agustín G. Martinelli, William Nava, Daniel J. Field

**Affiliations:** ^1^ Dinosaur Institute, Natural History Museum of Los Angeles, 900 Exposition Boulevard, Los Angeles, CA 90007, USA; ^2^ Department of Earth Sciences, University of Cambridge, Cambridge, UK; ^3^ Unidad de Paleontología, Departamento de Biología, Universidad Autónoma de Madrid, Madrid, Spain; ^4^ Sección Paleontología de Vertebrados, CONICET-Museo Argentino de Ciencias Naturales ‘Bernardino Rivadavia’, Buenos Aires, Argentina; ^5^ Museu de Paleontologia de Marília, Marília, São Paulo, Brazil; ^6^ Museum of Zoology, University of Cambridge, Cambridge, UK

**Keywords:** birds, brains, endocranium, dinosaurs, labyrinth, ear

## Abstract

Among terrestrial vertebrates, only crown birds (Neornithes) rival mammals in terms of relative brain size and behavioural complexity. Relatedly, the anatomy of the avian central nervous system and associated sensory structures, such as the vestibular system of the inner ear, are highly modified with respect to those of other extant reptile lineages. However, a dearth of three-dimensional Mesozoic fossils has limited our knowledge of the origins of the distinctive endocranial structures of crown birds. Traits such as an expanded, flexed brain, a ventral connection between the brain and spinal column, and a modified vestibular system have been regarded as exclusive to Neornithes. Here, we demonstrate all of these ‘advanced’ traits in an undistorted braincase from an Upper Cretaceous enantiornithine bonebed in southeastern Brazil. Our discovery suggests that these crown bird-like endocranial traits may have originated prior to the split between Enantiornithes and the more crownward portion of avian phylogeny over 140 Ma, while coexisting with a remarkably plesiomorphic cranial base and posterior palate region. Altogether, our results support the interpretation that the distinctive endocranial morphologies of crown birds and their Mesozoic relatives are affected by complex trade-offs between spatial constraints during development.

## 1. Introduction

The skull morphology of crown birds (Neornithes) is strikingly divergent from that of other extant reptiles [1,2]. Two major evolutionary transformations—the expansion of the premaxillae into the dominant component of the bony rostrum, and the acquisition of an enlarged and highly modified brain—reorganized the cranial architecture of crownward Mesozoic birds [3,4], overprinting the plesiomorphic dinosaurian condition. Over the last several decades, a wealth of Mesozoic fossil discoveries have greatly enriched our knowledge of the early evolution of the avian skull, in particular our understanding of the evolutionary reorganization of the facial [5–9], palatal [10–13], orbital [5,14] and temporal [5,6,9,15,16] regions. However, only a handful of these fossils preserve three-dimensional skulls that can provide detailed information about the origin and evolution of key cranial traits, including the nature of the brain and sensory systems. Three-dimensional preservation is essential for examining aspects of endocranial structure [17], including the morphology of the central nervous system, its connection with the spinal cord, and the morphology and associated function of sensory structures such as the inner ear, which houses the cochlea and the vestibular system (i.e. the organ of balance) [18]. These structures underpin the dazzling cognitive and sensory acuity of living birds, and some of these traits—a flexed brain connecting ventrally with the spinal cord and an enlarged, sinusoidal anterior semicircular canal of the vestibular system—are thought to have arisen very late in avian evolutionary history, only appearing along the most crownward portion of the avian phylogenetic tree [10,19]. However, the very few fossils that inform the early evolution of the avian endocranium are either very stemward long-tailed birds (e.g. Late Jurassic *Archaeopteryx*), whose endocranial anatomy is similar to non-avian theropods [20,21], or very crownward ornithurines close to the divergence of the crown group (e.g. Late Cretaceous *Ichthyornis* [6,10]), sharing many traits with living birds. Consequently, our understanding of the early evolution of the avian endocranium and brain is severely limited by the scarcity of three-dimensional skulls from Mesozoic birds, and the significant phylogenetic gap between taxa from which these data are available [22].

**Figure 1.**
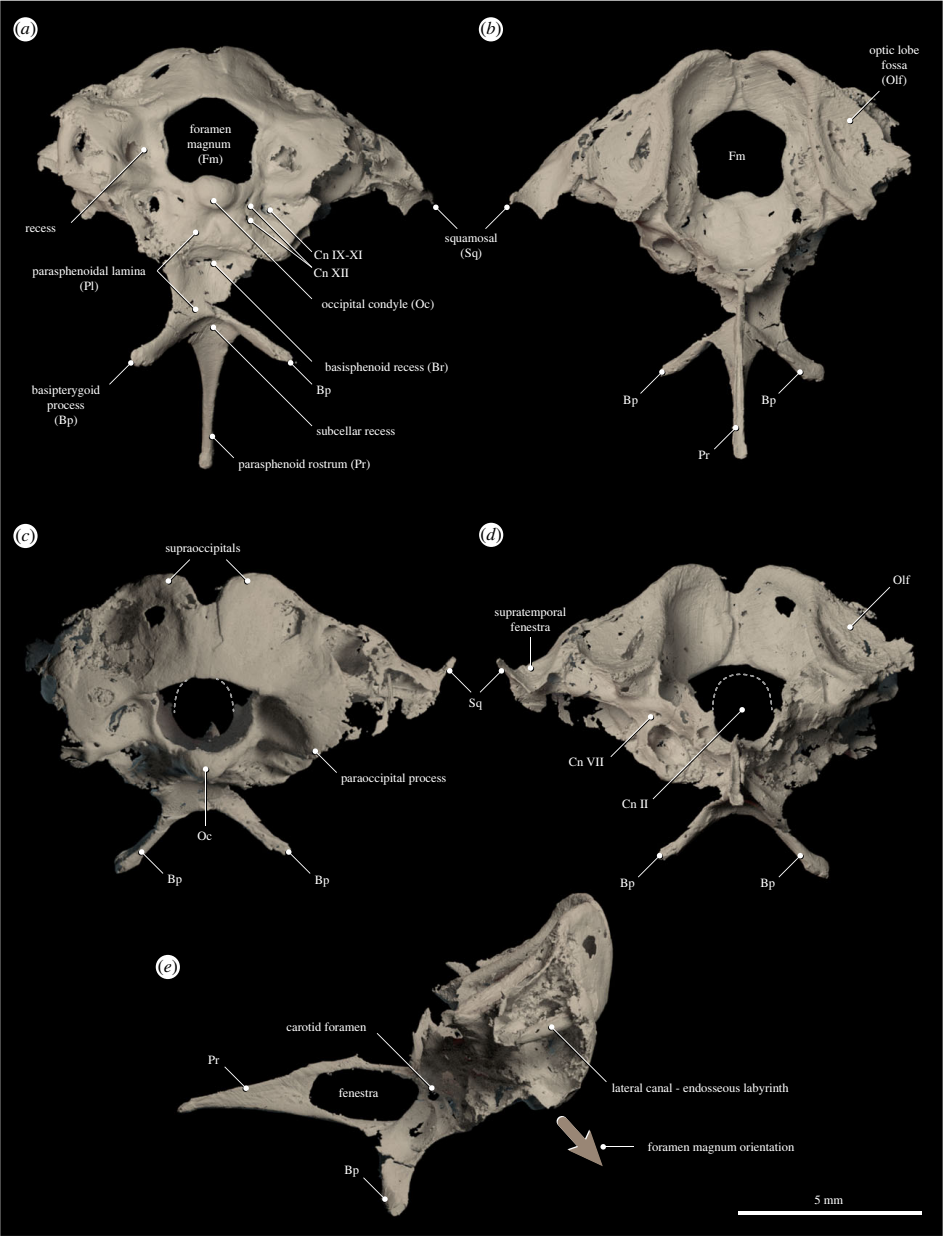
Enantiornithine braincase MPM-334-1 from the Late Cretaceous of southeastern Brazil. Three-dimensional meshes digitally rendered using Blender. (*a*) Ventral view, (*b*) dorsal view, (*c*) caudal view, (*d*) anterior view, and (*e*) left lateral view. Light brown arrow in (*e*) indicates orientation of the foramen magnum. Cn: exits of cranial nerves.

Here, we describe a partial braincase from an Upper Cretaceous (approx. 80 Ma) avian bonebed in southeastern Brazil (William's Quarry, Presidente Prudente, São Paulo State; Adamantina Formation, Bauru Group). The braincase is nearly undistorted (electronic supplementary material, figure S1; figure 1), and its three-dimensional preservation yields key new information on the endocranial anatomy of enantiornithines, a major clade of stem birds phylogenetically intermediate between *Archaeopteryx* and the most crownward portion of the avian stem lineage [23]. Despite an abundant, cosmopolitan fossil record that extends throughout most of the Cretaceous [24], the endocranial anatomy of enantiornithines remains completely unknown. By enabling, to our knowledge, the first detailed study of the endocranium of an enantiornithine, this new fossil substantially expands our knowledge of the early evolution of the avian braincase, brain and inner ear.

## 2. Results

MPM-334-1 (Museu de Paleontologia de Marília, São Paulo State) consists of a diminutive basicranium (approx. 1 cm between the occipital condyle and the tip of the parasphenoid rostrum; electronic supplementary material, figure S1; figure 1), falling within the size range of the smallest known enantiornithines, and some extant hummingbirds and small passerines [25]. The close association between MPM-334-1 and skeletal elements referable to enantiornithines (electronic supplementary material, figure S1), as well as the fact that, of nearly 1000 avian remains thus far recovered from William's Quarry, all diagnosable bones belong to Enantiornithes [26,27], support the identification of the new material as belonging to this major clade of stem birds. This identification is consistent with the morphology of the braincase, which exhibits traits observed in other enantiornithines (e.g. long, ventrally oriented basipterygoid processes known from *Zhouornis* [28]).

**Figure 2.**
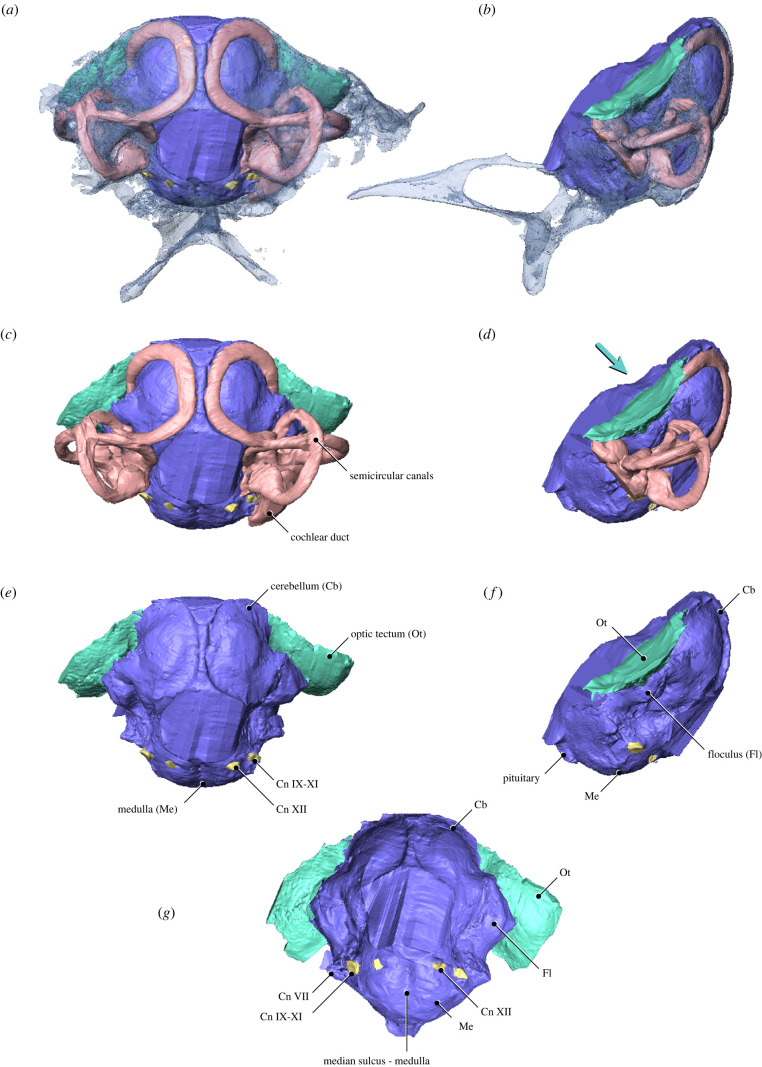
Main endocranial cavities of MPM-334-1. (*a*,*c,e*) caudal view, (*b*,*d*,*f*) left lateral view, and (*g*) ventral view. Teal arrow in (*d*) indicates the caudoventral direction in which the optic tectum pushes the anterior semicircular canal, arguably generating the characteristic crown bird-like ventral deflection in this structure. Cn: exits of cranial nerves.

The bones of the basicranium are fully fused with mostly no individualization (i.e. sutures) among the osteological components, similar to other adult or subadult enantiornithine specimens [28], suggesting that MPM-334-1 was skeletally mature (electronic supplementary material, figure S1; figure 1). The foramen magnum is subquadrangular, with a gently concave dorsal margin, and is much larger than the round occipital condyle, resembling the condition in the enantiornithine *Neuquenornis* [29] but unlike *Yuornis* [9] or *Zhouornis* in which these structures are more similar in size (electronic supplementary material, figure S1; figure 1). The orientation of the foramen magnum is nearly 60 degrees when the parasphenoid rostrum is oriented horizontally (the internal angle between the transverse plane of the foramen magnum and the longitudinal axis of the parasphenoid rostrum is 124.1 degrees; see Methods), indicating a substantial degree of braincase rotation (figure 1). The basipterygoid processes are long and slender and are directed ventrolaterally. The bases of these processes are connected at the midline to form a ridge separating the parasphenoidal lamina from the caudal end of the base of the parasphenoid rostrum. This ridge also separates a recessed area at the base (caudal end) of the parasphenoid rostrum (i.e. subcellar recess) from the recessed anterior portion of the parasphenoidal lamina (i.e. basisphenoid recess) (electronic supplementary material, figure S1; figure 1). The rostrally tapering parasphenoid rostrum bears a longitudinal furrow on its dorsal surface and is laterally perforated by a large oval fenestra (electronic supplementary material, figure S1). The parasphenoidal lamina is large and strongly recessed, most deeply near the basioccipital. In occipital view, there are deep circular recesses on either side of the foramen magnum; these are overhung laterally, dorsally and ventrally by projections of the exoccipital, forming a ventrolaterally projecting paraoccipital process (electronic supplementary material, figure S1; figure 1). The exit of a cranial nerve identified as the XII (hypoglossal, two exits as in *Neuquenornis* [29]) is visible, ventrally, on either side of the occipital condyle, which is also lateroventrally flanked by paired excavations (one on top of the other). Within the dorsally positioned excavation, preserved only on the right side, is an additional foramen, which we identify as the exit of a bundle of nerves including cranial nerves IX-XI (glossopharyngeal, vagus and accessory nerves) (figures 1 and 2). On the right side of the braincase, the caudal portion of a supratemporal fenestra appears to be preserved, formed by the caudal portion of the squamosal, incompletely fused to the remainder of the braincase (electronic supplementary material, figure S1; figure 1). While this region is poorly preserved, thus rendering this interpretation tentative, individualized squamosals have been reported for other enantiornithines [12].

The preserved endocast—visualized with micro-computed tomography (μ-CT) imaging (see Methods)—comprises impressions from the external surfaces of most of the medulla, parts of the cerebellum, and the ventral portion of the optic tectum (figure 2). We interpret the preserved right optic tectum fossa as mostly complete, capturing the ventral surface of this structure. The optic tectum lies immediately dorsal to the anterior semicircular canal of the endosseous labyrinth (figure 2) and is considerably more caudally positioned than in *Archaeopteryx*, *Cerebavis* and many crown birds [30]—the degree of caudal displacement is comparable to that of some extant hummingbirds (Neornithes: Trochilidae). The cerebellum appears not to be particularly expanded with respect to more crownward birds. In ventral view, the medulla is almost spherical and bears a medial sulcus. Ventral to the foramen for the optic nerve root there is a subrectangular protuberance which we interpret as the remnants of a small pituitary (figure 2). A few cranial nerve bundles are evident in the endocast. These include: (i) cranial nerve VII (facial), of which the exit is also visible in the braincase in cranial view and is positioned anteroventrally to the optic lobe fossa (figure 1); (ii) cranial nerve XII (hypoglossal), visible on each side ventrolateral to the foramen magnum; and (iii) cranial nerves IX-XI (glossopharyngeal, vagus and accessory nerves) (figure 1).

**Figure 3.**
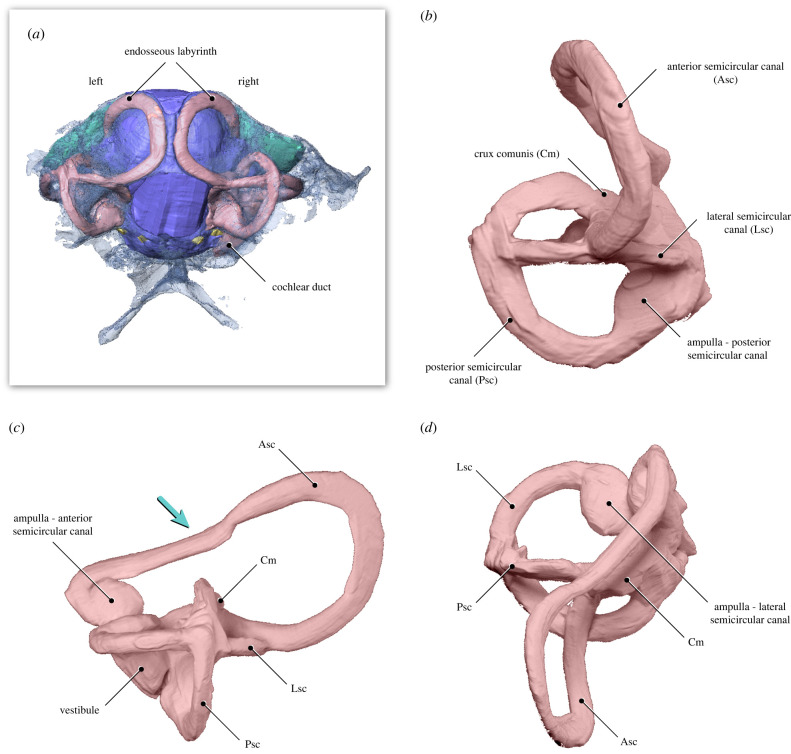
Anatomy of the endosseous labyrinth of the vestibular system and other inner ear structures of MPM-334-1. Teal arrow in (*c*) indicates the caudoventral deflection of the anterior semicircular canal of the endosseous labyrinth (caused by the displacement of the optic tectum, figure 2). (*a*) Inset displays the same view of the endocranial structures of MPM-334-1 displayed in figure 2*a*. (*b*–*d*) show different detailed views of the left endosseous labyrinth, oriented with its lateral semicircular canal completely horizontal (neutral Lsc). (*b*) Caudal (oriented to neutral Lsc), (*c*) lateral (oriented to neutral Lsc), and (*d*) dorsal (oriented to neutral Lsc). Note that the cochlear duct is not preserved on the left endosseous labyrinth but is visible on the right labyrinth (figure 2).

**Figure 4.**
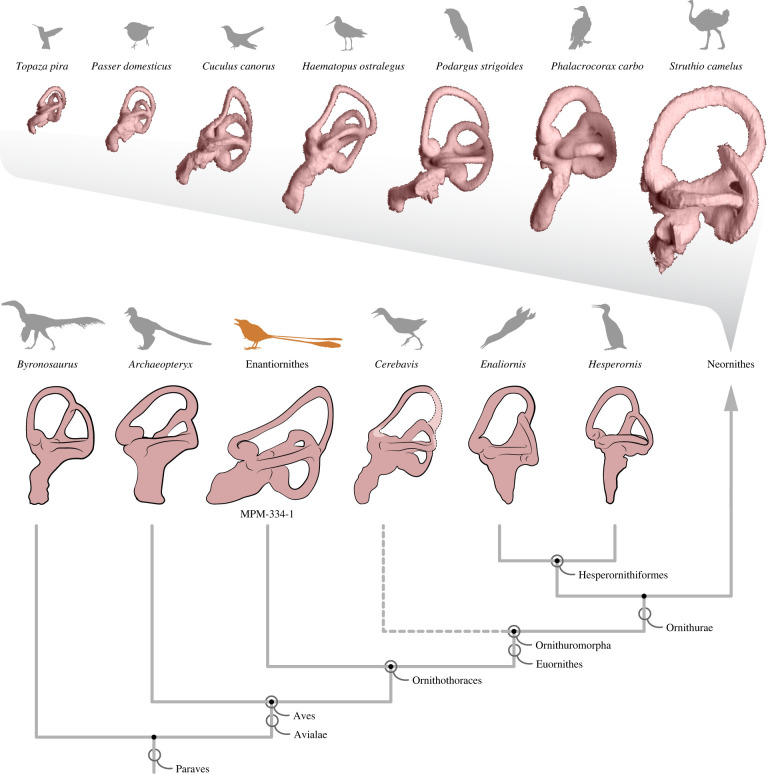
Comparative anatomy of the endosseous labyrinth and cochlear duct across selected birds (extinct and extant) and closely related non-avian dinosaurs. Labyrinths of extant birds sourced from [34]. *Byronosaurus*, *Archaeopteryx* and *Hesperornis* from [19]. *Cerebavis* from [32]. *Enaliornis* from [33]. Labyrinths are positioned following a neutral-cranial-base orientation for the whole crania (completely horizontal parasphenoid rostrum). Stem-based and node-based clade names are explicitly illustrated. Illustrations are not to scale.

**Figure 5.**
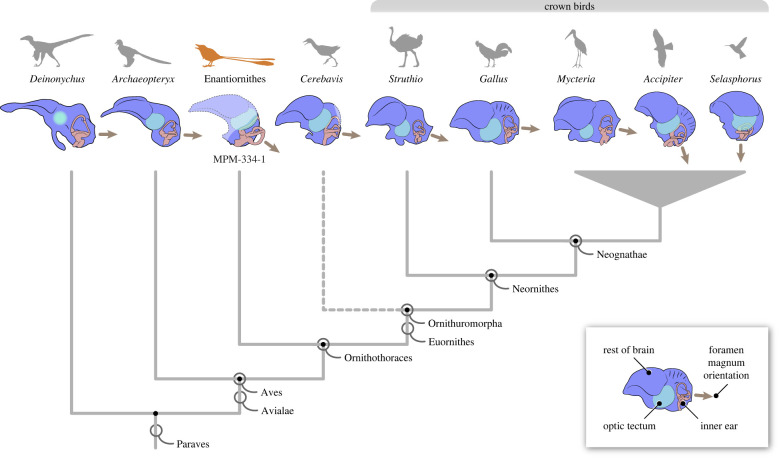
Main structures of central nervous system (including optic tectum) and inner ear (vestibular system and cochlear duct) across selected birds (extinct and extant) and closely related non-avian dinosaurs. While the orientation of the foramen magnum varies across phylogeny, the degree of ventralization of the foramen magnum in MPM-334-1 is significantly greater than that of other stem taxa, and comparable to that of certain crown birds (e.g. *Accipiter* and *Selasphorus*). Stem-based and node-based clade names are explicitly illustrated. Illustrations are not scaled.

The right and left inner ear cavities are almost completely preserved and are essentially undistorted (figures 2 and 3). The posterior and lateral semicircular canals of the endosseous labyrinth are circular and orthogonal to each other; they are subequal in length to one another and to a moderately expanded and ventromedially directed cochlear duct (only preserved on the right side) (figure 3). In cross-section, the canals are slightly flattened as in some crown birds (e.g. Anseriformes) [31]; this condition contrasts with the circular cross-section of the canals of *Archaeopteryx* [20] and *Cerebavis* [32]. The anterior semicircular canal is sinusoidal in shape and is expanded to approximately twice the length of the lateral and posterior canals (figures 1–3). It exhibits a dorsoventral deflection positioned ventral to the optic tectum, as in some extant birds (e.g. *Cuculus canorus* and *Haematopus ostralegus*) (figures 4 and 5). In Mesozoic birds, this deflection is absent in *Archaeopteryx*, *Enaliornis* and *Hesperornis*, and is only weakly developed in *Cerebavis* [19,20,32,33]. Caudally, the anterior semicircular canal has a ventrocaudal connection with the crux comunis near the level of the lateral canal (figure 3); this condition is comparable to that of some extant birds (e.g. *Haematopus ostralegus*) (figure 4), contrasting with other known Mesozoic birds, although this region is poorly preserved in *Cerebavis* [32]. The right and left anterior semicircular canals expand intraosseously towards the midline, approaching the level of the caudal midline of the cerebellum (figures 2 and 3). To our knowledge, such an extensive degree of medial expansion of the anterior semicircular canal has not been reported in any tetrapod, extinct or extant. The dimensions of the semicircular canals of MPM-334-1 fall towards the upper end of the spectrum of amniotes of similar cranial size and may indeed represent the largest known semicircular canals with respect to skull and endocast size yet reported.

## 3. Discussion

The new braincase (MPM-334-1) combines plesiomorphic dinosaurian traits (e.g. expanded, ventrolaterally facing basipterygoid processes and what appears to be a ‘diapsid’ supratemporal fenestra) with large semicircular canals and a flexed brain that are remarkably similar to those of extant birds (electronic supplementary material, figure S1; figures 1–4). In particular, the endosseous labyrinth of MPM-334-1 exhibits several traits only previously reported from the labyrinth of crown birds, such as an anterior semicircular canal with a prominent caudal extension and a marked ventral deflection of its cranial portion (figures 2–4).

**Figure 6.**
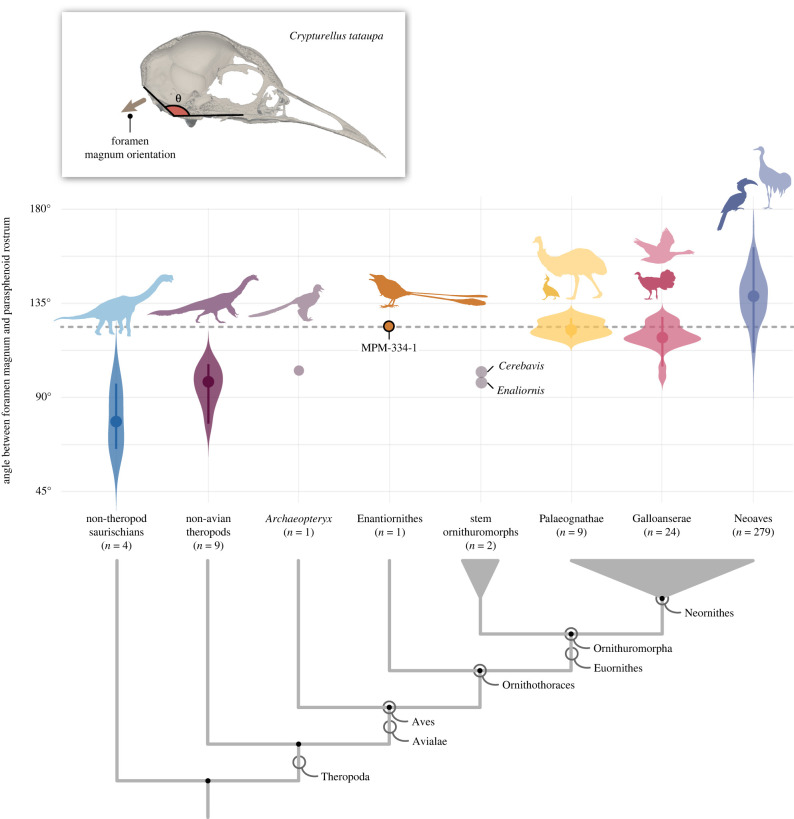
Orientation of the foramen magnum across birds and other saurischians. Violin plots show the distribution (contour) of the species values (large dot = mean value; line = interquartile ranges) for the angle between the foramen magnum and the parasphenoid rostrum (top left inset figure). Dashed line represents the angular value for MPM-334-1; *n* equals the number of specimens/species used for each taxon. Stem-based and node-based clade names are explicitly illustrated.

There is some evidence suggesting that the enlarged size of extant avian endosseous labyrinths is related to enhanced visual acuity [34], and that its size and marked ventral deflection is associated with enhanced manoeuvrability in some crown birds [19], although other investigations [34,35] have failed to support this latter interpretation. The enhanced flight proficiency of enantiornithines has been supported by different lines of evidence [36–39], and it is possible (as mentioned above) that some aspects of inner ear morphology may reflect adaptations for such aerial prowess [19,34,35]. Nonetheless, we argue the possibility that the crown-like vestibular traits observed in MPM-334-1 constitute an epiphenomenon related to brain flexion and the resultant caudal repositioning of the optic tectum need to be given careful attention. Specifically, we suggest that the ventral deflection of the anterior semicircular canal of MPM-334-1 may have been caused by the optic tectum pushing this enlarged canal downwards during skull development (figures 2–6). These same features are present in some crown birds in which the optic tectum (e.g. *Accipiter*) or other parts of the brain (e.g. the cerebrum in *Corvus*) appear to push the endosseous labyrinth ventrally during development (figure 5). Such mechanical effects acting during development can be observed in the post-hatching ontogeny of domestic fowl: in chicken hatchlings, the optic tectum lies close to the endosseous labyrinth, resulting in a marked ventral deflection of the anterior semicircular canal, yet this ventral deflection disappears during later stages of post-hatching development (i.e. the anterior semicircular canal becomes fully circular) as the optic tectum moves cranially, away from the endosseous labyrinth (fig. 4 in [40]).

**Figure 7.**
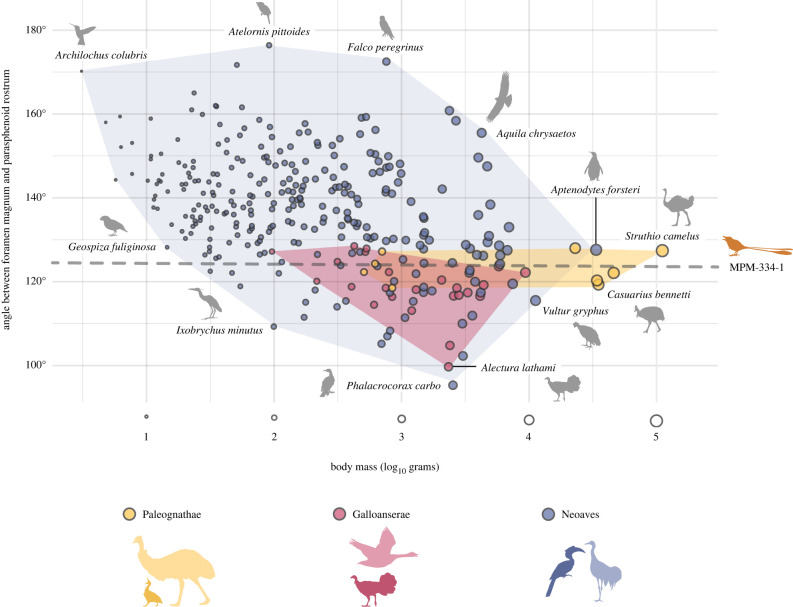
The relationship between orientation of the foramen magnum and body mass in crown birds. Dashed line indicates the angle for MPM-334-1. The convex hulls indicate the region encompassed by each major clade of crown birds (e.g. Palaeognathae, Galloanserae and Neoaves).

**Figure 8.**
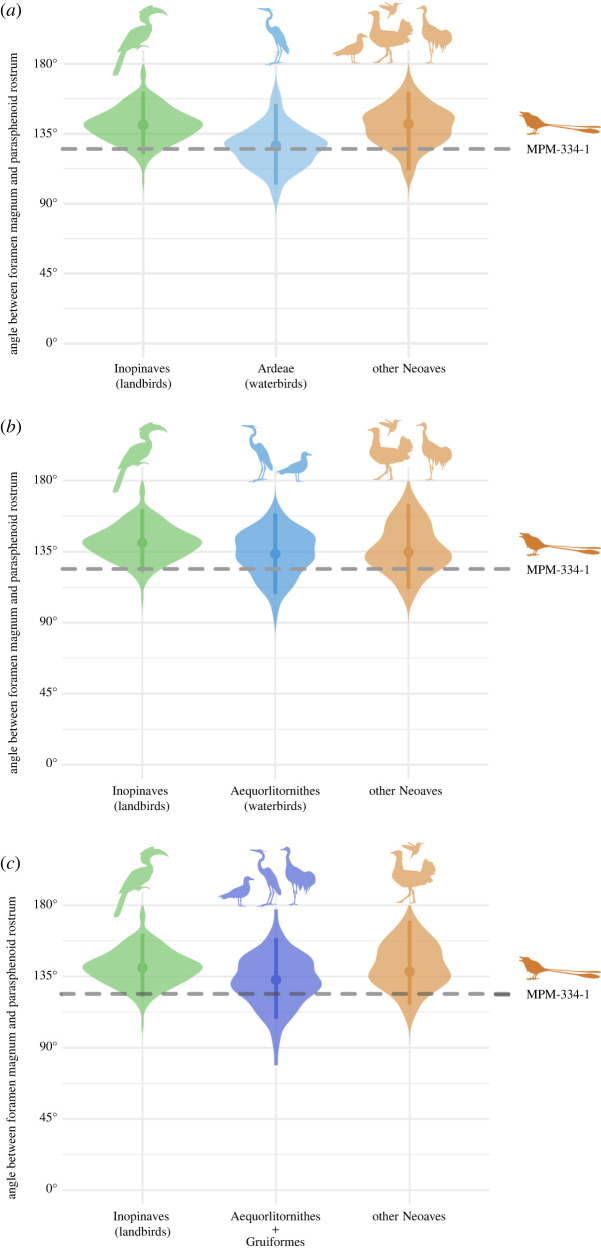
Orientation of the foramen magnum in Neoaves. Violin plots show the distribution (contour) of the species values (large dot = mean value; line = interquartile ranges) for the angle between the foramen magnum and the parasphenoid rostrum; dashed line indicates the angle for MPM-334-1. Three increasingly inclusive groups of neoavians with mostly aquatic ecologies are figured: (*a*) Ardeae; (*b*) Aequorlithornithes and an expanded grouping of water-linked clades; and (*c*) Aequorlithornithes + Gruiformes.

The repositioning of the optic tectum to a position dorsal to the endosseous labyrinth is one of the main consequences of the flexion of the brain observable in avian cranial endocasts (figures 3 and 4; [30]). In extant taxa in which the brain is unflexed (e.g. galliforms, cormorants, frogmouths and ostriches), the optic tectum and the endosseous labyrinth are positioned far apart from each other, and the anterior semicircular canal lacks a ventral deflection (figures 4 and 5). Additionally, in these taxa, the occiput tends to lack the ventralization characteristic of many other extant bird clades (e.g. Trochilidae, figure 5), suggesting that brain flexion is also closely linked to the orientation of the skull-neck joint. Prior to the discovery of MPM-334-1, a ventrally positioned foramen magnum had never been observed in any stem birds with three-dimensionally preserved skulls. Those observations led to the interpretation of occiput ventralization as an autapomorphy appearing within the avian crown group [10], and to hypotheses associating the ventralization of the occiput to the expansion and flexion of the brain, as well as enhanced aerial manoeuvrability [41,42]. In reality, avian foramen magnum orientation does not follow a binary distribution between a ventralized and non-ventralized position; instead, the position of the foramen magnum in crown birds encompasses a broad spectrum of angular values [43,44] (figures 5–8). Most importantly, by conducting a comprehensive comparison of the angle formed between the foramen magnum (and hence the occiput) and the parasphenoid rostrum of crown birds, we reveal that the degree of ventralization seen in MPM-344-1 falls within the range of variation of crown birds, significantly exceeding the values for taxa stemward of Ornithothoraces (figures 5 and 6). Surprisingly, this analysis demonstrates that MPM-334-1 also exhibits a greater degree of occiput ventralization than more crownward stem taxa (i.e. *Cerebavis* and *Enaliornis*), indicating either independent strong ventralization within enantiornithines or potential reversals to less ventralized morphologies along the line towards modern birds (reversals to a less ventralized morphology are also observed in certain crown bird subclades; figure 6).

Besides enhanced manoeuvrability, the primary determinants of occiput ventralization in birds are probably structural factors including body size (figure 7), orbit size [45], and the size of the brain and/or cranial base [46]. However, taxa belonging to several extant waterbird clades (Aequorlitornithes/Aequornithes) exhibit relatively non-ventralized occiputs, particularly when compared with the primarily terrestrial/arboreal landbird clade Inopinaves (figure 8) [46,47]. Both *Enaliornis* (a foot-propelled diver, [48,49]) and *Cerebavis* have been hypothesized to have exhibited water-linked ecologies, along with much of the avian stem lineage crownward of Enantiornithes [24], which could therefore underlie their lower degree of ventralization with respect to MPM-334-1.

Our observations suggest two alternative macroevolutionary scenarios in the history of avian endocranial evolution. On the one hand, the suite of endocranial transformations common to MPM-334-1 and many extant birds (i.e. a flexed brain, ventralized foramen magnum and ‘crown-like’ endosseous labyrinth) may have arisen among relatively small-bodied terrestrial and/or arboreal taxa prior to the origin of Ornithothoraces, while some crownward stem birds and neornithines (such as the aforementioned waterbirds) reverted to a condition resembling the plesiomorphic pre-ornithothoracine endocranium. On the other hand, an alternative scenario suggests that the same suite of endocranial features could represent homoplasies that arose multiple times throughout the evolutionary history of birds: within Enantiornithes such as in MPM-334-1, and again somewhere along the most crownward portion of the avian stem lineage (e.g. crownward of Hesperornithiformes). The latter scenario is consistent with the considerable degree of homoplasy already reported between enantiornithines and ornithuromorphs across the skeleton, including the independent acquisition of a rectricial feather fan [50], a toothless beak [9,13,51], similar patterns of bone fusion [52] and strongly carinated sterna [53]. Furthermore, supporting this ‘homoplastic’ scenario, a recently described cranial endocast from the near-crown bird *Ichthyornis* suggests that stem ornithuromorphs retained a plesiomorphically unflexed brain morphology until very late in their evolutionary history [10]. That said, it is important to keep in mind that some aspects of the aquatic ecology of *Ichthyornis* could underlie the unflexed nature of its brain as seen in many Aequornithes (see the above discussion).

**Figure 9.**
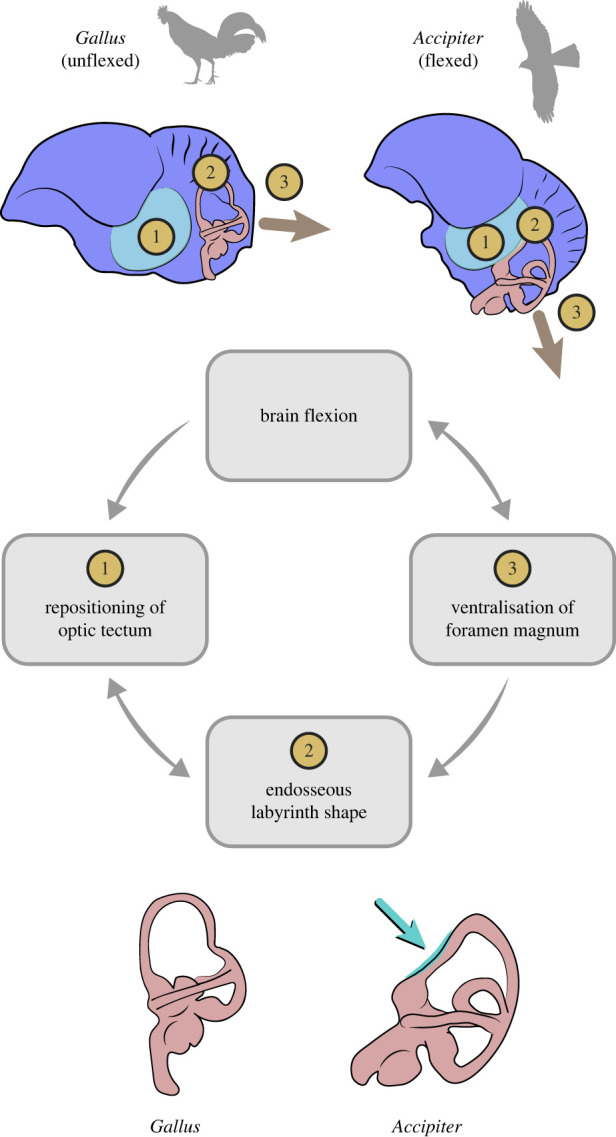
Conceptual diagram showing the non-flexed and flexed brain morphologies exhibited by extant birds, and the interplay of key hypothesized factors influencing the morphology of various systems within the avian cranium. Teal arrow pointing towards the labyrinth of *Accipiter* indicates the caudoventral deflection of the anterior semicircular canal of the endosseous labyrinth caused by the displacement of the optic tectum; figure 2.

Regardless of the precise phylogenetic origin of these traits among Mesozoic birds, our results and earlier work collectively suggest that the flexion of the avian brain is closely related to the orientation of the occiput [46], causing the repositioning of the optic tectum, and some aspects of the shape of the endosseous labyrinth [34] (figure 9). These observations agree with some postulations of the ‘Spatial Packing Hypothesis', which has been suggested to hold at broad phylogenetic scales in mammals (mainly primates, [54]), in birds [46], and to underlie endocranial similarities among pterosaurs and birds [35]. This hypothesis proposes that different systems within the head (e.g. eyes, brain and inner ear) compete for space within (and with) the neurocranium during development, highlighting the necessity of holistically considering the complex interplay among these components of the head (figures 7–9).

MPM-334-1 illustrates that a substantial degree of brain flexion and ventralization of the foramen magnum had already evolved among enantiornithine birds, indicating that important transformations towards a modern bird-like central nervous system and sensory organs arose early in avian evolutionary history. Conversely, other regions of the skull of MPM-334-1, especially the cranial base, retain a plesiomorphic dinosaurian configuration, illustrating that endocranial evolution in enantiornithines was somewhat disconnected from the morphological evolution of the remainder of the skull. Overall, the discovery of MPM-334-1 supports a more complex scenario for the evolution of the avian skull and central nervous system than had previously been understood, with key features of the modern avian endocranium evolving much earlier than what was formerly thought. Future discoveries of three-dimensionally preserved braincases from other Mesozoic birds, and their incorporation into quantitative analyses such as those presented here, will prove crucial for furthering our understanding of the origin and early evolution of the avian skull, brain and sensory systems.

## 4. Material and methods

### (a) Micro-computed tomography imaging

MPM-334-1 was scanned using a GE Phoenix Nanotom M at the Molecular Imaging Center of the University of Southern California (USC). MPM-334-1 was scanned at 9.99 µm voxel size, 125 kV, 200 mA, exposure time 750.36 ms, averaging two frames and skipping one frame, 360 degrees rotation 1440 frames and 0.1 mm Cu + Cu filter. The scans were initially reconstructed using GE phoenix datos|×2 2.3.3.160. The three-dimensional reconstruction of the skull was generated in Avizo Lite (9.2). Digital mesh cleaning was conducted using Geomagic (2013). Final imaging of the volumes was conducted using Blender and Avizo Lite (9.2).

### (b) Angular comparisons of foramen magnum orientation

To gain insight into the degree of ventralization of the foramen magnum in MPM-334-1, we measured the internal angle between the parasphenoid rostrum and the foramen magnum in the new specimen and compared it with a broad phylogenetic sample of skulls from non-avian dinosaurs, with a focus on near-avian theropods (*n* = 13), together with a large sample of crown and stem birds (*n* = 314) sourced from several online repositories (see the electronic supplementary material, table S1). We placed four landmarks on the three-dimensional meshes of the skull for each species: two within the parasphenoid rostrum and two at the dorsal-most and ventral-most points of the internal rim of the foramen magnum, using Stratovan Checkpoint (v.2019.03.04.1102) and its built-in functions to calculate the angle between the two vectors described by the four landmark coordinates.

## Data Availability

All angular comparisons are provided in the electronic supplementary material [55]. Three-dimensional digital mesh for the braincase MPM-334-1 and its endocranial structures and original μ-CT stack are available from the Dryad Digital Repository: https://doi.org/10.5061/dryad.79cnp5hzn [56].
